# Outcomes of non-metastatic HER-2 positive breast cancer before and after the introduction of anti-HER-2 therapy: a retrospective study from a tertiary Egyptian center

**DOI:** 10.1186/s43046-026-00420-4

**Published:** 2026-10-22

**Authors:** Aya Alaaeldin Youssef Ibrahim, Amal Mostafa Ismail, Mona Mohamed Fouda, Khaled Mahmoud Abouelkhair

**Affiliations:** Department of Clinical Oncology and Nuclear Medicine, Mansoura University Hospital, Mansoura, Egypt

**Keywords:** Breast cancer, HER-2 positive, Anti-HER-2 therapy, Disease-free survival, Pathological complete response, Overall survival

## Abstract

**Background:**

HER-2 positive breast cancer accounts for 15–20% of cases and is associated with aggressive behavior and poor prognosis. Anti-HER-2 therapy, including trastuzumab and pertuzumab, has improved outcomes in Western populations; however, real-world data in Egyptian patients remain limited. This study evaluated pathological response, disease-free survival (DFS), and overall survival (OS) among Egyptian patients with non-metastatic HER-2 positive breast cancer treated before and after the introduction of anti-HER-2 therapy.

**Methods:**

We retrospectively analyzed 200 female patients with non-metastatic HER-2 positive breast cancer treated in two treatment eras: before the introduction of anti-HER-2 therapy (2014–2016, *n* = 100) and after its introduction (2020–2022, *n* = 100). Patients in the later era received trastuzumab alone or trastuzumab plus pertuzumab in the neoadjuvant or adjuvant setting. Outcomes included pathological complete response (pCR), DFS, OS. Survival outcomes were assessed using Kaplan–Meier analysis and compared using the log-rank test. Univariable and multivariable Cox proportional hazards regression analyses were performed for DFS and OS.

**Results:**

Anti-HER-2 therapy was associated with a higher pCR rate among patients who received neoadjuvant treatment (40.0% vs. 10.3%, *p* < 0.001) and more favorable postoperative tumor and nodal staging. The median follow-up duration was 51.2 months in the non-anti-HER-2 group and 40.0 months in the anti-HER-2 group. During follow-up, disease recurrence occurred in 60% of patients in the non-anti-HER-2 group compared with 17% in the anti-HER-2 group (*p* < 0.001), while overall mortality was 13% in the anti-HER-2 group compared with 59% in the non-anti-HER-2 group (*p* < 0.001). Multivariable Cox regression analysis showed that anti-HER-2 therapy was associated with improved DFS (HR 0.274, 95% CI 0.108–0.697; *P* = 0.007) and OS (HR 0.260, 95% CI 0.093–0.728; *P* = 0.010).

**Conclusion:**

In this retrospective study of Egyptian patients with non-metastatic HER-2 positive breast cancer, treatment in the later era, when anti-HER-2 therapy was available, was associated with higher pathological response rates and improved DFS and OS. However, given the historical-cohort design and differences in baseline characteristics and treatment strategies between the two eras, these findings cannot establish a causal effect of anti-HER-2 therapy alone. Larger multicenter studies are warranted to further evaluate outcomes and address treatment-era and selection-related confounding in the Egyptian setting.

## Introduction

Breast cancer is the most common malignancy among women worldwide and a leading cause of cancer-related mortality, with approximately 2.3 million new cases and over 680,000 deaths reported in 2020 [1, 2].

In Egypt, breast cancer is the most common female malignancy, representing nearly 40% of all female cancers. Many patients are diagnosed at advanced stages [3, 4]. This is due to low screening participation and limited awareness compared with high-income countries [5, 6].

HER-2 positive tumors account for 15–20% of breast cancers and are characterized by aggressive tumor biology, high cellular proliferation, and historically worse clinical outcomes compared with other breast cancer subtypes [7, 8]. The introduction of anti-HER-2 therapies, particularly trastuzumab and pertuzumab, has markedly improved the prognosis [9]. Trastuzumab, a monoclonal antibody directed against the HER-2 receptor, was the first targeted therapy shown to significantly improve survival outcomes in both early and metastatic disease [10]. Subsequent clinical trials have demonstrated additional benefits with dual HER-2 blockade using trastuzumab and pertuzumab [11].

Landmark randomized clinical trials, including HERA, NSABP B-31, and NCCTG N9831, have demonstrated significant improvements in survival outcomes when anti-HER-2 therapy is combined with chemotherapy [12–14]. Despite these advances, real-world data on treatment outcomes among Egyptian patients with HER-2 positive breast cancer remain relatively limited.

Therefore, this study aimed to evaluate pathological complete response (pCR), disease-free survival (DFS), and overall survival (OS) among patients with non-metastatic HER-2 positive breast cancer treated before and after the introduction of anti-HER-2 therapy.

## Methods

### Study design and population

This retrospective study was conducted at our institution and included 200 female patients with non-metastatic HER-2 positive breast cancer. Patients were divided into two groups according to treatment era: pre anti-HER-2 therapy era **(**2014–2016, *n* = 100) and post anti-HER-2 therapy era (2020–2022, *n* = 100).

#### Inclusion criteria

Patients were eligible if they met the following criteria:

- Female patients aged ≥ 18 years.
- Pathologically confirmed non-metastatic HER-2 positive breast cancer.
- HER-2 positivity confirmed by immunohistochemistry (IHC 3+) or IHC 2 + with positive silver in situ hybridization (SISH).
- Patients who underwent surgery either before or after neoadjuvant treatment.

### Data collection

Clinical and pathological data were obtained from medical records and included age, menopausal status, family history, comorbidity, ECOG performance status, tumor stage, nodal status, histological subtype, tumor grade, hormone receptor status (ER and PR), Ki-67 proliferation index, lymphovascular invasion, perineural invasion, type of surgery, chemotherapy regimen and timing, postoperative radiotherapy, and anti-HER-2 therapy.

### Outcomes

The primary outcomes were pCR, DFS, and OS. Disease-free survival was defined as the time from the date of surgery to the date of first documented disease recurrence; for patients without recurrence, the date of last follow-up was used. Overall survival was defined as the time from the date of diagnosis to the date of death or the date of last follow-up for patients who remained alive.

### Statistical analysis

Statistical analysis was performed using SPSS software version 26. Continuous variables were expressed as means ± standard deviation, while categorical variables were presented as frequencies and percentages. Survival outcomes were evaluated using Kaplan–Meier survival analysis, and comparisons between groups were performed using the log-rank test. Cox proportional hazards regression analysis was performed for both DFS and OS. Univariable and multivariable Cox regression analyses were conducted, and hazard ratios (HRs) with 95% confidence intervals (CIs) and corresponding P values were reported. The multivariable models included demographic characteristics, baseline disease characteristics, tumor characteristics, and treatment-related variables available in the study dataset, including age, menopausal status, family history, comorbidity, baseline tumor stage, tumor grade, HER-2 assessment, ER status, PR status, Ki-67, lymphovascular invasion, perineural invasion, type of surgery, type and timing of chemotherapy, postoperative radiotherapy, anti-HER-2 therapy, timing of anti-HER-2 therapy, pathological response, and pathological stage. Given the retrospective design and the comparison of two different treatment eras, the findings were interpreted as associations and not as evidence of a causal effect of anti-HER-2 therapy. A P value < 0.05 was considered statistically significant.

## Results

### Patient characteristics

The mean age was 50.79 ± 11.05 years in the non-anti-HER-2 group and 48.12 ± 10.85 years in the anti-HER-2 group (*p* = 0.086). Menopausal status, marital status, and family history were similar between the groups. Cardiac disease was more frequent in the non-anti-HER-2 group (11% vs. 1%, *p* = 0.003), whereas ECOG performance status 0 was more frequent in the anti-HER-2 group (25% vs. 13%, *p* = 0.009) (Table 1).

**Table 1:** Baseline patient characteristics (*N* = 200)

Patient Characteristic	No anti-HER-2 (*n* = 100)	Anti-HER-2 (*n* = 100)	*P* value
Age (years), mean ± SD	50.79 ± 11.05	48.12 ± 10.85	0.086
Menopausal status			1.000
Premenopausal	52 (52%)	52 (52%)	
Postmenopausal	48 (48%)	48 (48%)	
Marital status			1.000
Married	99 (99%)	99 (99%)	
Single	1 (1%)	1 (1%)	
Family history of cancer			0.208
Negative	76 (76%)	68 (68%)	
Positive	24 (24%)	32 (32%)	
Cardiac disease			0.003*
No	89 (89%)	99 (99%)	
Yes	11 (11%)	1 (1%)	
ECOG performance status			0.009*
PS 0	13 (13%)	25 (25%)	
PS 1	87 (87%)	71 (71%)	
PS 2	0 (0%)	4 (4%)	

Data are presented as mean ± standard deviation (SD) or number (%). *ECOG *Eastern Cooperative Oncology Group performance status

*p < 0.05 indicates statistical significance

### Disease characteristics

Clinical T stage (*p* = 0.575), overall clinical stage (*p* = 0.100), and tumor grade (*p* = 0.351) were comparable between the groups. However, clinical nodal involvement differed significantly between the groups (*p* = 0.016), with a higher proportion of N3 disease in the anti-HER-2 group (11% vs. 1%). Invasive ductal carcinoma predominated in both groups (95% and 98%, respectively). ER positivity was 63% in the anti-HER-2 group versus 55% in the non-anti-HER-2 group, while PR positivity was 61% versus 55%, respectively. HER-2 assessment differed significantly between the groups, with IHC 2+/SISH+ cases being more frequent in the non-anti-HER-2 group (17% vs. 3%, *p* = 0.001). High Ki-67 expression (≥ 20%) was more frequent in the anti-HER-2 group (84.6% vs. 61%, *p* = 0.003). Lymphovascular and perineural invasion were not significantly different between the groups (*p* = 0.269 and *p* = 0.127, respectively) (Table 2).

**Table 2:** Baseline tumor and clinicopathological characteristics (*N* = 200)

Tumor Characteristic	No anti-HER-2 (*n* = 100)	Anti-HER-2 (*n* = 100)	*P* value
Clinical T-stage			0.575
Tx / Tis	1 (1%)	0 (0%)	
T1	17 (17%)	19 (19%)	
T2	69 (69%)	64 (64%)	
T3	10 (10%)	10 (10%)	
T4	3 (3%)	7 (7%)	
Clinical N-stage			0.016*
Nx / N0	32 (32%)	25 (25%)	
N1	67 (67%)	63 (63%)	
N2	0 (0%)	1 (1%)	
N3	1 (1%)	11 (11%)	
Baseline clinical stage			0.100
Stage IA	9 (9%)	8 (8%)	
Stage IIA	29 (29%)	27 (27%)	
Stage IIB	49 (49%)	43 (43%)	
Stage IIIA	9 (9%)	7 (7%)	
Stage IIIB	3 (3%)	4 (4%)	
Stage IIIC	1 (1%)	11 (11%)	
Tumor grade			0.351
G1	2 (2%)	1 (1%)	
G2	71 (71%)	63 (63%)	
G3	27 (27%)	36 (36%)	
Histology			0.425
IDC	95 (95%)	98 (98%)	
ILC	1 (1%)	0 (0%)	
Special type	4 (4%)	2 (2%)	
ER status			0.250
Negative	45 (45%)	37 (37%)	
Positive	55 (55%)	63 (63%)	
PR status			0.390
Negative	45 (45%)	39 (39%)	
Positive	55 (55%)	61 (61%)	
HER-2 assessment			0.001*
IHC 3+	97 (97%)	83 (83%)	
IHC 2+ / SISH+	3 (3%)	17 (17%)	
Ki-67 (*n* = 132)			0.003*
<20%	16 (39%)	14 (15.4%)	
≥20%	25 (61%)	77 (84.6%)	
LVI (*n* = 95)			0.269
Negative	6 (16.2%)	15 (25.9%)	
Positive	31 (83.8%)	43 (74.1%)	
PNI (*n* = 39)			0.127
Negative	5 (50%)	22 (75.9%)	
Positive	5 (50%)	7 (24.1%)	

Data are presented as n (%). *IDC* invasive ductal carcinoma, *ILC* invasive lobular carcinoma, *ER *estrogen receptor, *PR* progesterone receptor, *HER-2* human epidermal growth factor receptor 2, *IHC* immunohistochemistry, *SISH* silver in situ hybridization, *LVI* lymphovascular invasion, *PNI* perineural invasion. Ki-67, LVI, and PNI data were available only for a subset of patients. *p < 0.05 indicates statistical significance

### Treatment characteristics

Surgery after neoadjuvant therapy was more frequent in the anti-HER-2 group (60% vs. 31%), whereas frontline surgery was more common in the non-anti-HER-2 group (69% vs. 40%, *p* < 0.001). Breast-conserving surgery was more common in the anti-HER-2 group (33% vs. 18%), while modified radical mastectomy was more frequent in the non-anti-HER-2 group (82% vs. 67%, *p* = 0.015).

All patients in the anti-HER-2 group received anthracycline- and taxane-based chemotherapy. In the non-anti-HER-2 group, 78% received combined anthracycline- and taxane-based regimens, 20% received anthracycline-based chemotherapy alone, and 2% received no chemotherapy (*p* < 0.001). The timing of chemotherapy also differed significantly between the groups, with neoadjuvant chemotherapy administered to 60% of patients in the anti-HER-2 group compared with 29.6% in the non-anti-HER-2 group, while adjuvant chemotherapy was administered to 40% and 70.4%, respectively (*p* < 0.001). Postoperative radiotherapy was more frequently administered in the anti-HER-2 group than in the non-anti-HER-2 group (93% vs. 75%, *p* = 0.001).

Among patients in the anti-HER-2 group, 75% received trastuzumab alone and 25% received trastuzumab plus pertuzumab. Anti-HER-2 therapy was administered in the neoadjuvant setting in 25% of patients and in the adjuvant setting in 75% (Table 3).

**Table 3:** Treatment characteristics

Treatment variable	No anti-HER-2 (*n* = 100)	Anti-HER-2 (*n* = 100)	*P* value
Time of surgery			< 0.001
Frontline surgery	69 (69%)	40 (40%)	
Post-NAC	31 (31%)	60 (60%)	
Type of surgery			0.015
BCS	18 (18%)	33 (33%)	
MRM	82 (82%)	67 (67%)	
Type of chemotherapy			< 0.001
No chemotherapy	2 (2%)	0 (0%)	
Anthracycline-based	20 (20%)	0 (0%)	
Anthracycline + taxane	78 (78%)	100 (100%)	
Timing of chemotherapy (*n* = 198)			< 0.001
Neoadjuvant	29 (29.6%)	60 (60.0%)	
Adjuvant	69 (70.4%)	40 (40.0%)	
PORT			0.001
No	25 (25%)	7 (7%)	
Yes	75 (75%)	93 (93%)	
Type of anti-HER-2 therapy			< 0.001
None	100 (100%)	0 (0%)	
Trastuzumab	0 (0%)	75 (75%)	
Trastuzumab + Pertuzumab	0 (0%)	25 (25%)	
Timing of anti-HER-2 therapy			—
Neoadjuvant	—	25 (25%)	
Adjuvant	—	75 (75%)	

Data are presented as number (%). *BCS *breast-conserving surgery, *MRM* modified radical mastectomy, *NAC* neoadjuvant chemotherapy, *PORT* post-operative radiotherapy

### Pathological response

Pathological response was assessed in 89 patients who received neoadjuvant treatment, including chemotherapy with or without anti-HER-2 therapy. Among these patients, the pCR rate was higher in the anti-HER-2 group than in the non-anti-HER-2 group (40.0% vs. 10.3%, *p* < 0.001). Tumor and lymph-node down-staging were also more frequent in the anti-HER-2 group (*p* < 0.001). These findings were accompanied by a higher proportion of patients achieving earlier pathological stages at the time of surgery (Table 4). Given the retrospective nature of the study and the comparison of treatment groups from different periods, these findings were interpreted descriptively and should not be attributed solely to anti-HER-2 therapy.

**Table 4:** Pathological outcomes

Pathological outcome	No anti-HER-2	Anti-HER-2	*p*-value
Pathological response (*n* = 89)			< 0.001
pCR	3 (10.3%)	24 (40.0%)	
Partial response	6 (20.7%)	21 (35.0%)	
No response	20 (69.0%)	15 (25.0%)	
Pathological T-stage (*n* = 200)			< 0.001
Tx / Tis	6 (6%)	31 (31%)	
T1	9 (9%)	15 (15%)	
T2	63 (63%)	42 (42%)	
T3	16 (16%)	10 (10%)	
T4	6 (6%)	2 (2%)	
Pathological N-stage (*n* = 200)			< 0.001
N0 / Nx	19 (19%)	46 (46%)	
N1	29 (29%)	32 (32%)	
N2	22 (22%)	15 (15%)	
N3	30 (30%)	7 (7%)	
Overall pathological stage (*n* = 200)			< 0.001
Stage 0	3 (3%)	24 (24%)	
Stage IA	4 (4%)	10 (10%)	
Stage IB	0 (0%)	5 (5%)	
Stage IIA	15 (15%)	19 (19%)	
Stage IIB	21 (21%)	13 (13%)	
Stage IIIA	24 (24%)	20 (20%)	
Stage IIIB	3 (3%)	2 (2%)	
Stage IIIC	30 (30%)	7 (7%)	

Data are presented as number (%). *pCR* pathological complete response

### Survival outcomes

**Fig. 1:**
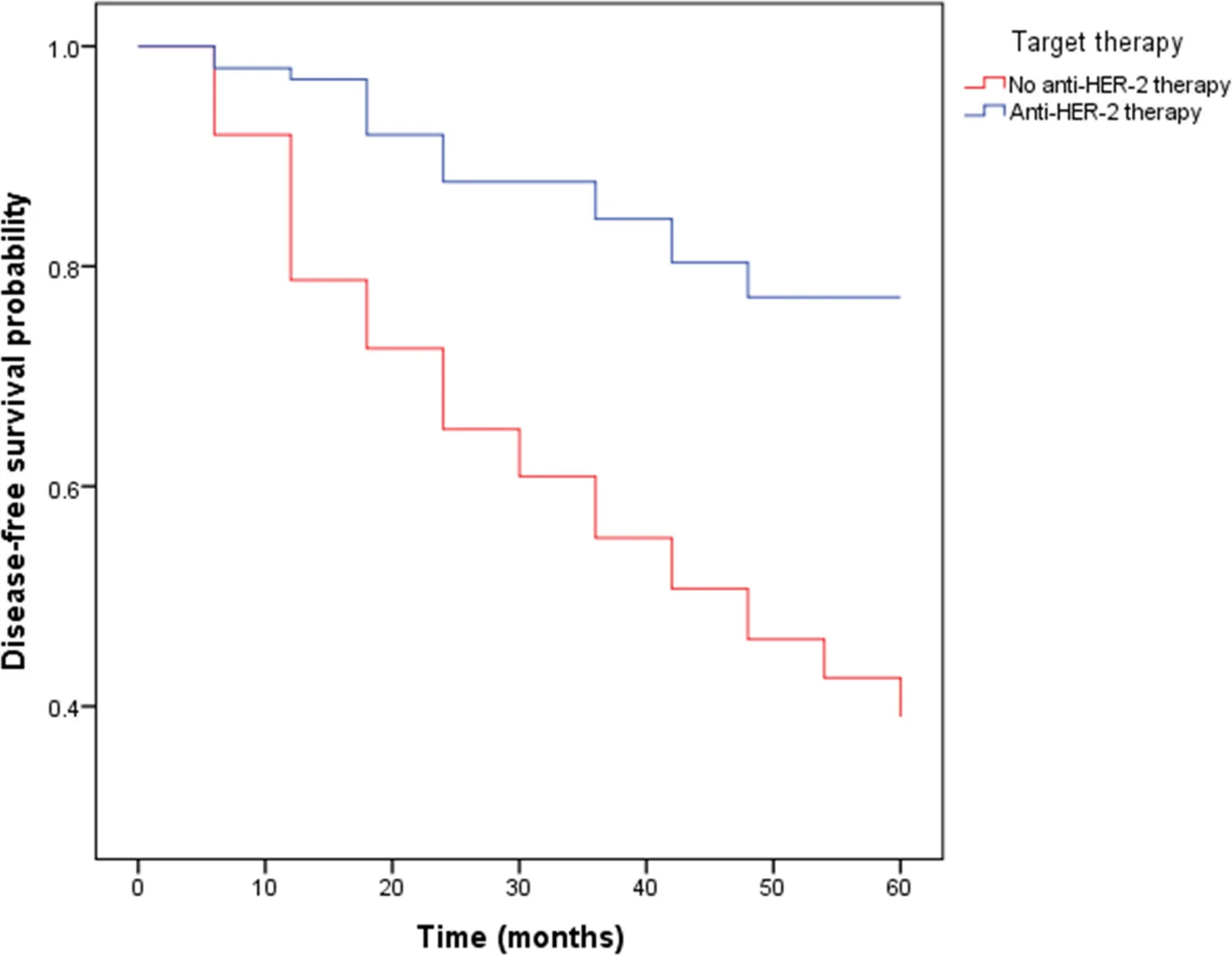
Kaplan–Meier curve showing DFS according to anti-HER-2 therapy (60-month analysis). The difference between the two groups was statistically significant (log-rank *p* < 0.001)

The median follow-up duration was 51.2 months in the non-anti-HER-2 group and 40.0 months in the anti-HER-2 group. During the follow-up period, disease recurrence occurred in 60% of patients in the non-anti-HER-2 group compared with 17% in the anti-HER-2 group (*p* < 0.001). Kaplan–Meier analysis showed longer DFS among patients who received anti-HER-2 therapy. The median DFS was 54.0 months in the anti-HER-2 group compared with 42.9 months in the non-anti-HER-2 group (*p* < 0.001) (Fig. 1; Table 5).

**Fig. 2:**
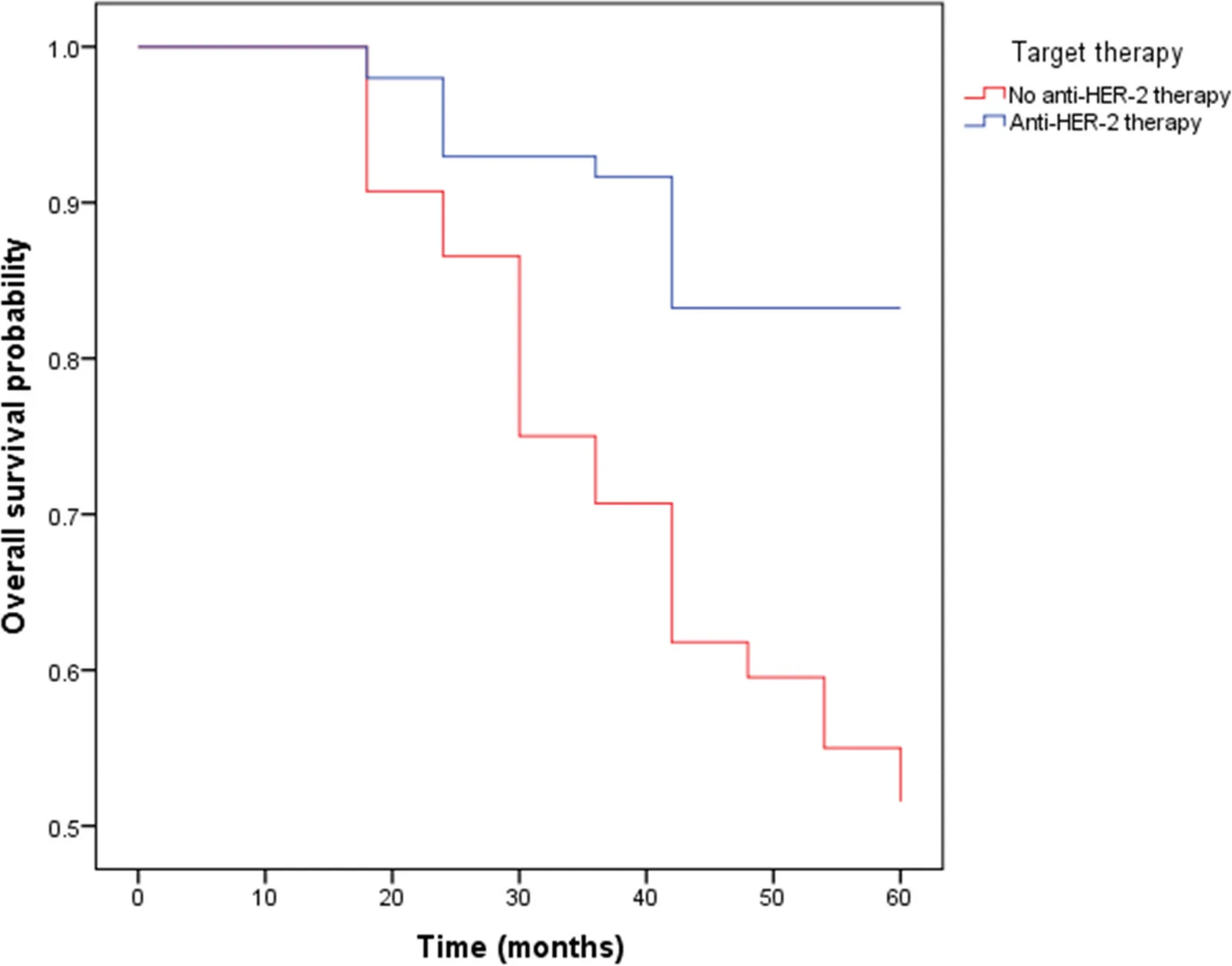
Kaplan–Meier curve showing OS according to anti-HER-2 therapy (60-month analysis). The difference between the two groups was statistically significant (log-rank *p* < 0.001)

Overall survival was also significantly better in the anti-HER-2 group. Overall mortality during follow-up was 13% in the anti-HER-2 group compared with 59% in the non-anti-HER-2 group (*p* < 0.001). Kaplan–Meier analysis showed longer OS among patients who received anti-HER-2 therapy (Fig. 2; Table 5).

**Table 5:** Clinical outcomes during follow-up

Outcome	No anti-HER-2 (*n* = 100)	Anti-HER-2 (*n* = 100)	*P* value
Mortality	59 (59%)	13 (13%)	< 0.001
Recurrence	60 (60%)	17 (17%)	< 0.001

Data are presented as number (%)

### Cox regression analysis of factors associated with DFS

**Fig. 3:**
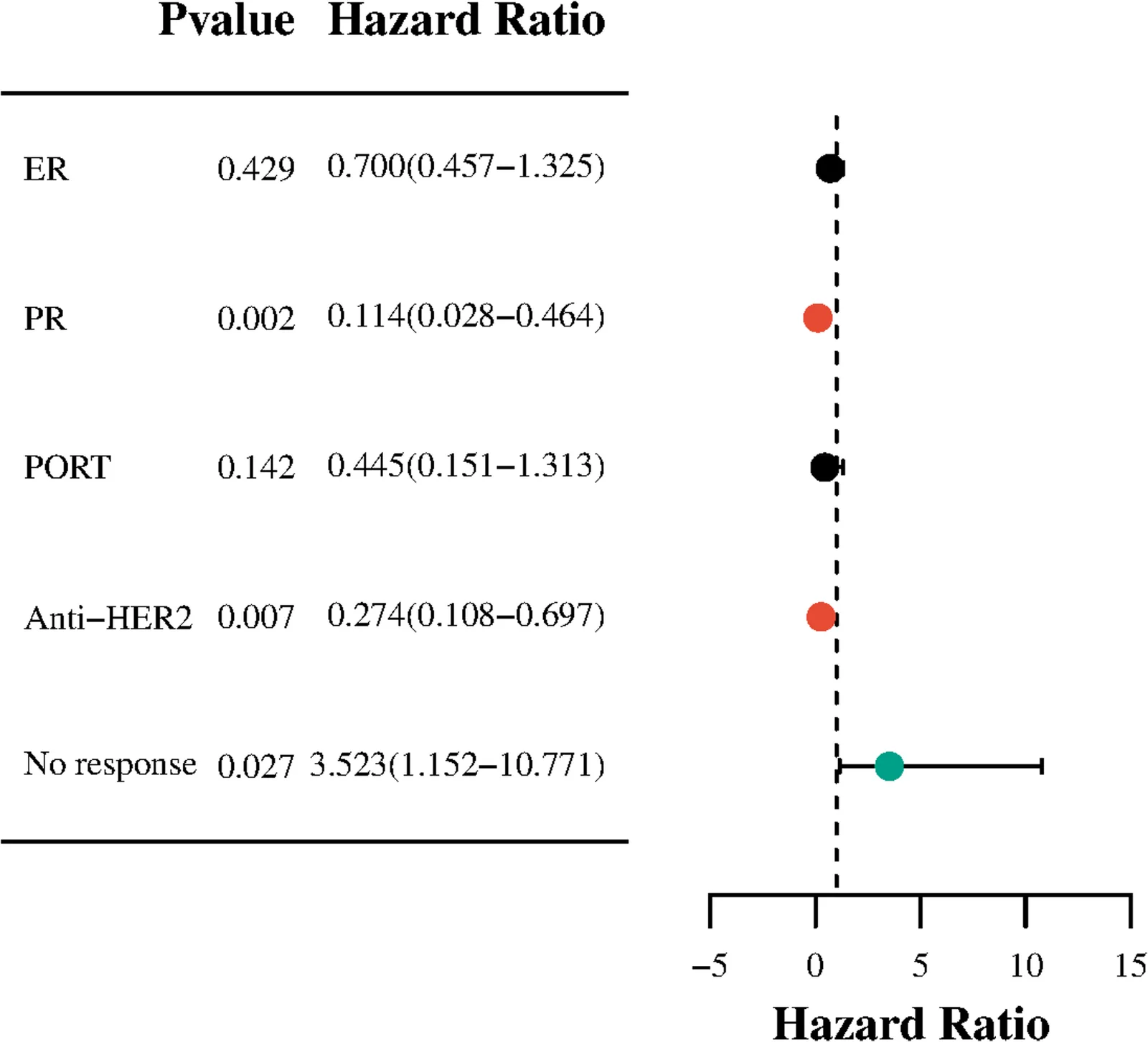
Forest plot of multivariable Cox regression analysis for DFS

Univariable and multivariable Cox regression analyses were performed to identify factors associated with DFS. In univariable analysis, positive ER status, positive PR status, postoperative radiotherapy, and anti-HER-2 therapy were significantly associated with longer DFS, whereas absence of pathological response was associated with shorter DFS. In the multivariable analysis, positive PR status and anti-HER-2 therapy remained significantly associated with DFS, while absence of pathological response remained associated with a higher hazard of recurrence or death. Anti-HER-2 therapy was associated with a lower hazard of recurrence or death (HR 0.274, 95% CI 0.108–0.697; *P* = 0.007) (Table 6; Fig. 3).

**Table 6:** Cox regression analysis of factors associated with DFS

Variables	Univariable			Multivariable		
	*P* value	HR	95% CI	*P* value	HR	95% CI
Age	0.891	0.999	0.978–1.020	—	—	—
Menopausal status (postmenopausal vs. premenopausal)	0.976	1.007	0.642–1.579	—	—	—
Family history (Positive vs. negative)	0.237	0.726	0.428–1.234	—	—	—
Comorbidity (yes vs. no)	0.810	1.060	0.657–1.713	—	—	—
Baseline Tumor stage (advanced vs. early)	0.154	1.429	0.875–2.333	—	—	—
Tumor grade						
Grade 2 vs. Grade 1 Grade 3 vs. Grade 1	0.460 0.754	2.108 1.381	0.291–15.265 0.184–10.341	—	—	—
ER status (positive vs. negative)	< 0.001	0.349	0.221–0.550	0.429	0.700	0.457–1.325
PR status (positive vs. negative)	< 0.001	0.314	0.198–0.499	0.002	0.114	0.028–0.464
HER-2 (IHC 3 + vs. IHC 2+/SISH+)	0.745	0.870	0.377–2.008	—	—	—
Ki-67 (≥ 20% vs. < 20%)	0.169	0.613	0.305–1.232	—	—	—
LVI (positive vs. negative)	0.741	1.179	0.445–3.122	—	—	—
PNI (positive vs. negative)	0.367	0.488	0.103–2.320	—	—	—
Type of surgery (MRM vs. BCS)	0.138	1.534	0.871–2.702	—	—	—
Type of chemotherapy						
Anthracycline-based vs. no chemotherapy Anthracycline + taxane vs. no chemotherapy	0.546 0.728	1.869 0.704	0.245–14.275 0.097–5.101	—	—	—
Timing of chemotherapy (adjuvant vs. neoadjuvant)	0.085	0.664	0.416–1.059	—	—	—
PORT (positive vs. negative)	< 0.001	0.159	0.097–0.262	0.142	0.445	0.151–1.313
Anti-HER-2 therapy (positive vs. negative)	< 0.001	0.299	0.172–0.517	0.007	0.274	0.108–0.697
Timing of anti-HER-2 (adjuvant vs. neoadjuvant)	0.154	4.381	0.576–33.330	—	—	—
Pathological response						
Partial response vs. pCR No response vs. pCR	0.415 0.009	1.593 3.709	0.520–4.882 1.393–9.875	0.027	3.523	1.152–10.771
Pathological staging						
Early vs. non-invasive Advanced vs. non-invasive	0.462 0.113	1.456 2.106	0.536–3.954 0.838–5.296	—	—	—

*HR* hazard ratio, *CI* confidence interval, *ER* estrogen receptor, *PR* progesterone receptor, *HER-2* human epidermal growth factor receptor 2, *IHC* immunohistochemistry, *SISH* silver in situ hybridization, *LVI* lymphovascular invasion, *PNI* perineural invasion, *MRM* modified radical mastectomy, *BCS* breast-conserving surgery, *PORT* postoperative radiotherapy, *pCR* pathological complete response. *P* < 0.05 indicates statistical significance

### Cox regression analysis of factors associated with OS

**Fig. 4:**
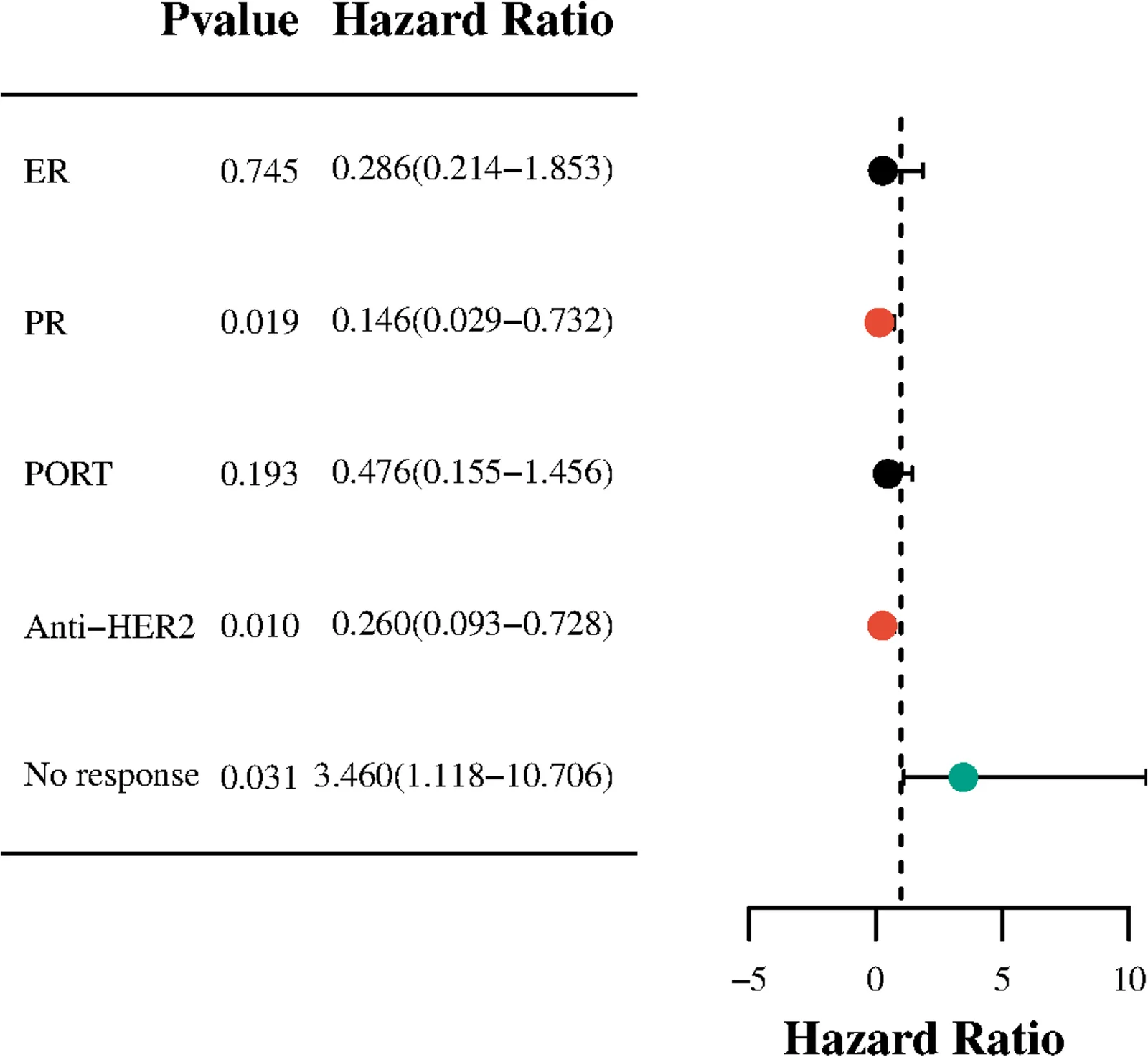
Forest plot of multivariable Cox regression analysis for OS

Univariable and multivariable Cox regression analyses were performed to identify factors associated with OS. In univariable analysis, positive ER status, positive PR status, postoperative radiotherapy, and anti-HER-2 therapy were significantly associated with longer OS, whereas absence of pathological response was associated with shorter OS. In the multivariable analysis, positive PR status and anti-HER-2 therapy remained significantly associated with OS, while absence of pathological response remained significantly associated with a higher hazard of death. Anti-HER-2 therapy was associated with a lower hazard of death (HR 0.260, 95% CI 0.093–0.728; *P* = 0.010) (Table 7; Fig. 4).

**Table 7:** Cox regression analysis of factors associated with OS

Variables	Univariable			Multivariable		
	*P* value	HR	95% CI	*P* value	HR	95% CI
Age	0.511	1.007	0.985–1.030	—	—	—
Menopausal status (postmenopausal vs. premenopausal)	0.712	1.092	0.685–1.741	—	—	—
Family history (Positive vs. negative)	0.258	0.718	0.405–1.274	—	—	—
Comorbidity (yes vs. no)	0.501	1.183	0.726–1.928	—	—	—
Baseline Tumor stage (advanced vs. early)	0.359	1.266	0.765–2.094	—	—	—
Tumor grade						
Grade 2 vs. Grade 1 Grade 3 vs. Grade 1	0.506 0.681	1.959 1.526	0.270-14.208 0.203–11.474	—	—	—
ER status (positive vs. negative)	< 0.001	0.307	0.190–0.496	0.745	0.286	0.214–1.853
PR status (positive vs. negative)	< 0.001	0.305	0.188–0.494	0.019	0.146	0.029–0.732
HER-2 (IHC 3 + vs. IHC 2+/SISH+)	0.864	1.083	0.434–2.705	—	—	—
Ki-67 (≥ 20% vs. < 20%)	0.634	0.831	0.386–1.785	—	—	—
LVI (positive vs. negative)	0.966	0.979	0.363–2.640	—	—	—
PNI (positive vs. negative)	0.444	0.539	0.111–2.624	—	—	—
Type of surgery (MRM vs. BCS)	0.113	1.658	0.888–3.097	—	—	—
Type of chemotherapy						
Anthracycline-based vs. no chemotherapy Anthracycline + taxane vs. no chemotherapy	0.667 0.859	1.561 0.835	0.205–11.899 0.115–6.070	—	—	—
Timing of chemotherapy (adjuvant vs. neoadjuvant)	0.069	0.633	0.386–1.037	—	—	—
PORT (positive vs. negative)	< 0.001	0.197	0.121–0.322	0.193	0.476	0.155–1.456
Anti-HER-2 therapy (positive vs. negative)	< 0.001	0.313	0.168–0.585	0.010	0.260	0.093–0.728
Timing of anti-HER-2 (adjuvant vs. neoadjuvant)	0.297	2.976	0.383–23.103	—	—	—
Pathological response						
Partial response vs. pCR No response vs. pCR	0.485 0.019	1.506 3.261	0.477–4.751 1.213–8.768	0.031	3.460	1.118–10.706
Pathological staging						
Early vs. non-invasive Advanced vs. non-invasive	0.755 0.234	1.177 1.757	0.423–3.274 0.694–4.443	—	—	—

*HR* hazard ratio, *CI* confidence interval, *ER* estrogen receptor, *PR* progesterone receptor, *HER-2* human epidermal growth factor receptor 2, *IHC* immunohistochemistry, *SISH* silver in situ hybridization, *LVI* lymphovascular invasion, *PNI* perineural invasion, *MRM* modified radical mastectomy, *BCS* breast-conserving surgery, *PORT* postoperative radiotherapy, *pCR* pathological complete response. *P* < 0.05 indicates statistical significance

### Subgroup survival analysis

Kaplan–Meier analyses were performed to explore the association between anti-HER-2 therapy and DFS and OS according to ER status, tumor grade, and Ki-67 proliferation index.

Among ER-negative patients, anti-HER-2 therapy was associated with longer DFS (*p* < 0.001) and OS (*p* < 0.001). Among ER-positive patients, the association was not statistically significant for either DFS (*p* = 0.076) or OS (*p* = 0.105).

Among patients with grade II and III tumors, anti-HER-2 therapy was associated with longer DFS (grade II, *p* < 0.001; grade III, *p* = 0.018) and OS (grade II, *p* = 0.003; grade III, *p* = 0.009). Analysis of grade I tumors was limited by the small number of events.

Among patients with high Ki-67 expression (≥ 20%), anti-HER-2 therapy was associated with longer DFS (*p* = 0.004), while the association with OS was of borderline statistical significance (*p* = 0.050). No statistically significant differences were observed among patients with low Ki-67 expression (< 20%). These subgroup findings were exploratory, as formal interaction testing was not performed to determine whether the association between anti-HER-2 therapy and survival differed significantly across subgroups.

## Discussion

In this retrospective study, we evaluated the clinical outcomes of Egyptian patients with non-metastatic HER-2 positive breast cancer treated before and after the introduction of anti-HER-2 therapy. Patients treated in the later era, when anti-HER-2 therapy was available, had higher pathological response rates and longer DFS and OS. These patients also experienced markedly lower recurrence and mortality rates. These findings are consistent with results from major randomized trials, including the HERA trial and the NSABP B-31/N9831 studies, which demonstrated that the addition of trastuzumab to standard chemotherapy significantly reduces the risk of recurrence and improves survival in patients with HER-2 positive breast cancer [12, 13]. However, as this was a retrospective study comparing two different treatment eras, and given the differences in baseline characteristics and treatment strategies between the groups, these findings should be interpreted as an association rather than as evidence of a causal effect of anti-HER-2 therapy alone.

The higher use of neoadjuvant systemic therapy in the anti-HER-2 group (2020–2022) reflects differences in treatment strategies between the two treatment eras. The greater use of neoadjuvant therapy in the later era may have contributed to the observed differences in pathological response and survival outcomes and therefore represents a potential confounding factor when comparing the two groups.

In addition to survival outcomes, our study demonstrated a significant increase in pCR rates among patients receiving anti-HER-2 therapy. Achieving pCR after neoadjuvant therapy is widely recognized as an important prognostic indicator associated with improved long-term survival [15]. These findings are also consistent with previous neoadjuvant trials that reported higher response rates with anti-HER-2 therapy [16]. Pathological response was assessed in 89 patients who received neoadjuvant treatment, including neoadjuvant chemotherapy with or without anti-HER-2 therapy. Therefore, the pCR analysis was limited to this subgroup. In addition, the use and composition of neoadjuvant treatment differed between the two treatment eras; therefore, the higher pCR rate should be interpreted cautiously and cannot be attributed solely to anti-HER-2 therapy.

Subgroup analyses suggested that the association between anti-HER-2 therapy and improved survival was observed across several clinicopathological subgroups, including patients with different tumor grades, ER status, and Ki-67 proliferation index. The association between treatment-era groups and survival was statistically significant among patients with ER-negative disease, whereas the corresponding associations among patients with ER-positive disease did not reach statistical significance; however, these findings should be considered exploratory, as formal interaction testing was not performed.

Several limitations should be considered. First, the retrospective design introduces the possibility of selection bias and residual confounding. Second, the two treatment groups represented different treatment eras, with differences in treatment strategies, particularly the use and timing of neo adjuvant therapy, type of surgery, and postoperative radiotherapy. Although these treatment-related variables were considered in the multivariable analysis, residual confounding related to the treatment era and other unmeasured factors cannot be excluded. Third, differences in follow-up duration between the two treatment eras may influence survival comparisons. In addition, pathological response was assessed only among patients who received neo adjuvant treatment, limiting the pCR analysis to this subgroup. Finally, the study was conducted at a single center, which may limit generalizability.

Despite these limitations, our findings are broadly consistent with the established evidence supporting anti-HER-2 therapy in HER-2 positive breast cancer. Our results provide real-world evidence of improved pathological response and survival outcomes among patients treated in the later treatment era, while recognizing that differences in treatment strategies and other temporal factors may have contributed to the observed outcomes. These findings highlight the potential importance of access to anti-HER-2 therapy as part of curative treatment strategies for Egyptian patients with HER-2 positive breast cancer in routine clinical practice.

## Conclusion

In this retrospective study of Egyptian patients with non-metastatic HER-2 positive breast cancer, treatment in the later era, when anti-HER-2 therapy was available, was associated with higher pathological response rates and longer DFS and OS. However, given the historical-cohort design and differences in baseline characteristics and treatment strategies between the two eras, these findings cannot establish a causal effect of anti-HER-2 therapy alone. Larger multicenter studies are warranted to further evaluate outcomes and address treatment -era and selection-related confounding in the Egyptian setting.

## Data Availability

All data supporting the findings of this study are available within the paper.

## Abbreviations

BCS: breast-conserving surgery
CI: confidence interval
DFS: disease-free survival
ECOG: Eastern Cooperative Oncology Group
ER: estrogen receptor
HER-2: human epidermal growth factor receptor 2
HR: hazard ratio
IHC: immunohistochemistry
IDC: invasive ductal carcinoma
ILC: invasive lobular carcinoma
LVI: lymphovascular invasion
MRM: modified radical mastectomy
NAC: neoadjuvant chemotherapy
OS: overall survival
pCR: pathological complete response
PNI: perineural invasion
PORT: postoperative radiotherapy
PR: progesterone receptor
SISH: silver in situ hybridization
